# Percutaneous nephrolithotomy in new versus those patients with previous history of Extracorporeal Shock Wave Lithotripsy on ipsilateral side

**DOI:** 10.12669/pjms.38.4.5116

**Published:** 2022

**Authors:** Nadeem Iqbal, Sajid Iqbal, Nasir Zareen, Keron Akintola Ayodele Blair

**Affiliations:** 1 Nadeem Iqbal, Shifa International Hospital, Islamabad, Pakistan; 2 Sajid Iqbal, Department of Rehabilitation, Pakistan Navy PNS Hospital, Karachi, Pakistan; 3 Nasir Zareen, SKBZ/CMH Rawlakot; 4 Keron Akintola Ayodele Blair, American International School of Medicine, Georgetown Guyana

**Keywords:** PCNL, Stone Free Rates, Shock Wave Lithotripsy, Renal Stones, Complications

## Abstract

**Objectives:**

To appraise the effects of previous history of ineffectual extracorporeal shockwave lithotripsy (SWL) treatment on the execution and end result of percutaneous nephrolithotomy (PCNL).

**Methods:**

The study was performed from January 2012 till November 2019 at the urology department at our hospital, a tertiary healthcare center. In total, four hundred and twenty two patients were enrolled in the study. We arranged the study participants into two groups. Group-I comprised of 66 subjects who had undergone failed SWL 12 months prior to PCNL procedure, while Group-II included patients who had no history of previous SWL. Information related to study variables was registered in designated proformas and then processed in SPSS version 16 for the statistical computations.

**Results:**

On the whole, the mean age of patients was 45.25± 14.38 years. While the mean calculus size was 494.80±128.83 mm^2^. The complexity of stones formulated on the basis of Guy’s stone score was identical among the two groups. American Society of Anesthesiologists (ASA) class categories were almost similar among the two groups. Stone free rates of 80.30% and 81.74% (p value=0.73) were observed in Group-I and II respectively. Time to create PCNL tract and mean drop in hemoglobin were noted to be significantly higher in Group-I. Complication rates and grades were not being dissimilar among the two groups.

**Conclusion:**

Patients having prior history of unsuccessful SWL history before undertaking the PCNL procedure manifested similar stone free rates and complications rates as those observed in SWL naive cases of PCNL.

## INTRODUCTION

ESWL is benchmark treatment for smaller renal stones (size below 2 cm). While PCNL is employed for larger calculi (complex and size more than 2 cm).^1^-^4^ The average cost of ESWL per treatment has made the procedure very much affordable to people with moderate income.^4^-^6^ This led to misuse of SWL in last few years especially in less developed regions and non academic hospitals. As indicated by the recent researches, some factors lead to lower stone free rates in patients having longer skin to stone distance, higher BMI, stone Hounsfield units and lower pole location. So, patients with history of previously failed SWL are encountered more frequently in urology practice nowadays.^5^,^6^

Repeated use of SWL contributes to changes such as fibrous degeneration and contortion of collecting system.^7^-^12^ Up till now there are only few studies in literature that have thrown light on the ramifications of previous failed and frequent SWL procedures on the outcomes and complications of succeeding PCNL. Focus of present study was to share experience regarding the PCNL outcomes in those patients who had failed prior SWL sessions in a developing world country.

## METHODS

The study was performed from January 2012 till November 2019 at the urology department at our hospital, after the approval of institutional review board and ethical committee (IRB#936-210-2017).

Patients showing features of congenital renal anomaly, ureteropelvic junction obstruction, age less than 18 years, those having abnormal coagulation profile, active urinary tract infection, and those who failed to come to come for follow up were excluded from the study. Additionally, those without CT scan studies prior to the PCNL procedure had to be excluded from the present study. Thus in total, four hundred and twenty two patients were enrolled in the study.

All patients undertook non-contrast CT scan for stones estimation preceding PCNL. Additionally, complete blood count, serum electrolytes, renal function tests, coagulation evaluation, urine routine analysis and culture tests, coagulation study were conducted. Stones complexity was allotted by utilization of Guy’s stone score (GSS). Relevant antibiotic treatment with antibiotics was administered to patients, who were positive for bacterial colonies on urine cultures preoperatively. PCNL was executed by standard technique in the prone position.

### PCNL procedure

Triangular or Bull’s eye technique was utilized according to case to case. Lower pole was preferred to get an entry to the calyx. Contrast assisted opacification of collecting system was utilized to aid in having proper and safe entry of 18 G needle into the desired place within the kidney. Then over the wire, increasing size fascial dilators were utilized. Later on, Alken metallic dilators were pushed over the olive tip for tract creation. Following this, an Amplatz sheath (24-30 fr) was slid into the created tract and nephroscopy was carried out to locate and then retrieve the stones in the kidney. Pneumatic Lithoclast was applied for stone disintegration. Stone grasper (three-prong grasper) helped in stone retrieval. After that insertion of a 6 fr DJ stent was done in an antegrade fashion. The nephrostomy tube was secured with silk thread.

### Follow up of Patients

At three months in outdoor clinic with help of Ultrasound and X-Ray KUB to look for status of any residual stones. The detection of residual fragments of size ≤ 4 mm or no fragments at all on radiology assessment was labelled as success of therapy.

### Statistical Analysis

Analysis was achieved by utilizing SPSS version 16. Application of Mean along with standard deviation values was utilized in case of the continuous variables. While frequency/percentages portrayed categorical factors. We took help of independent student’s t-test for comparing the continuous factors, while to weigh up categorical values between the groups, Chi-square test was utilized. A p value of <0.05 was judged as statistically crucial value.

## RESULTS

In entirety 422 subjects were taken in the final analysis. Total of 66 patients were kept in Group-I that comprised of subjects having previous history of failed shock wave lithotripsy (SWL). While, 356 patients in Group-II were those who had no previous history of SWL. On the whole, the mean age of patients was 45.25±14.38 years. While the mean calculus size was 494.80±128.83 mm^2^. The complexity of stones formulated on the basis of Guy’s stone score was identical among the two groups (Table-I). The overall mean procedure time was almost 135 minutes and was not much dissimilar among the two groups (Table-II). Mean time for access to pelvicalyceal system was 13.12±4.99 minutes in Group-I as compared to 11.49±3.86 minutes in Group-II (P value=0.003, Table-II). Stone-free rate was relatively higher among the SWL naïve patients (Group-II), however no significant difference was seen among the two groups in terms of stone free rates and residual stones.Table-II. Mean fall in hemoglobin was 1.68±0.59 mg/dl in first group in contrast to 1.42±0.85mg/dl in second (p value=0.021, Table-II).

**Table-I:** Demographic variables.

	Group-I (Previous history of SWL)	Group-II (No history of SWL)	P-value
Number of patients	66	356	---
Mean Age	45.17±14.08 years	45.13±14.91 years	0.98
Male	44 (66.6%)	254 (71.3%)	0.463
Female	22 (33.4%)	102 (28.6%)	
Right Renal stone	27 (40.9%)	145 (40.7%)	0.97
Left Renal stone	39 (59.1%)	211 (59.3%)	
Body Mass Index	27.79±3.39	26.68±4.48	0.057
Mean stone size (mm^2^)	469.77±72.51 mm^2^	499.45±136.31 mm^2^	0.086
***Guys Stone Score***			
Guys Stone Score 1	32 (48.48%)	171 (48.03%)	0.46
Guys Stone Score 2	23 (34.84%)	129 (36.23%)	
Guys Stone Score 3	10 (15.15%)	38 (10.67%)	
Guys Stone Score 4	1 (1.51%)	18 (5.05%)	
***ASA Class***			
ASA Class 1	49 (74.24%)	274 (76.96%)	0.77
ASA Class 2	13 (19.69%)	67 (18.82%)	
ASA Class 3	4 (6.06%)	15 (4.21%)	

**Table-II:** Details of Procedure Outcomes.

	Group-I (Previous history of SWL)	Group-II (No history of SWL)	P-value
Stone free rate	53 (80.30%)	291(81.74%)	0.73
Residual stones	13 (19.69%)	65 (18.26%)	0.73
Mean Operative time	139.21±56.86 minutes	134.83±71.18 minutes	0.63
Number of tracts	1.15±0.36	1.11±0.31	0.36
Time to create tract	13.12±4.99 minutes	11.49±3.86 minutes	0.003
Nephrostomy tube	43/66 (65.15%)	206/356 (57.86%)	0.46
Drop in hemoglobin	1.68±0.59 mg/dl	1.42±0.85mg/dl	0.021
Hospital stay	2.94±0.79 days	2.99±1.27 days	0.77

Complications were described in line with Clavien-Dindo Classification. Majority of the complications fell in Clavien-Grade-1. Major complications such as iatrogenic renal vessel injury, or death were not encountered in any of the groups (Table-III).

**Table-III:** Complications.

Complication Grade*	Type Complication	Group-I	Group-II	p-value
1	Fever	5 (7.57%)	20 (5.61%)	0.56
1	Illeus	2 (3.03%)	7 (1.96%)	0.63
1	Pelvicalyceal puncture (extravasation)	2 (3.03%)	12 (3.37%)	1.0
2	Transfusion need	4 (6.06%)	13 (3.65%)	0.31
2	Sepsis	1 (1.51%)	5 (1.40%)	1.0
3	Perinephric abscess	1 (1.51%)	2 (0.56%)	0.40
3	Iatrogenic bowel injury	0%	0%	---
3	Iatrogenic Renal vascular injury (angioembolisation)	0%	0%	
4	Septic Shock (need for ICU management)	0 %	1 (0.28%)	1.0
5	Death	0 %	0%	---

* Clavien-Dindo classification

## DISCUSSION

Shock wave lithotripsy has fewer complications. The immediate common complications when they occur pertain to the mechanical damage from the shock wave. The shock wave can blow out the fragile arcuate veins proceeding to interstitial hemorrhage and hematoma formation. Apart from this, interstitial fibrosis occurs with segmental shrinkage of renal cortex.^11^-^13^ In addition to these changes, pressure waves (when ESWL procedures are applied frequently), can thrust the stone fragments in the neighborhood of renal mucosa. Yuruk et al.^10^ noted, shrank calyceal infundibulum and submerged stone chips underneath the pelvicalyceal mucosal layer in patients who earlier undertook sessions of failed SWL. Similarly, Wen Zhong et al found that Stone fragments submerged within renal tissue and narrowed calyceal infundibulum expanded the likelihood of residual stones after PCNL surgery. They had an inferior net stone clearance in the failed SWL group than in the patients who did not have antecedent intervention.^11^

Resorlu B et al. noticed no significant difference (in patients with antecedent open surgery or failed SWL history) regarding operative duration (minutes), time needed to acquire entrance to the renal collecting system, duration of fluoroscopy, adverse outcomes of PCNL and hospitalization duration.^9^ Yuruk E et al. observed comparable operative and fluoroscopic imaging duration in their study groups.^10^ Operative time was similar in two groups in the present study as well (Table-II). They attained a success rate approximating 89%. Their procedural success and frequency of complications were alike among the two groups. We had the similar success rates among the two groups (Table-II). They had increased operative and fluoroscopic imaging duration per cm2 of stone secondary to the tissue reaction of SWL and dispersed calculi fragments within the renal pelvicalyceal system.

Zhong W et al. studied sixty-two patients (who had failed SWL history). The average duration between SWL and PCNL procedures approximated 6.48 months.^11^ They remarked that mean time to acquire a renal pelvicalyceal access was close among their groups (10.5 vs. 9.6 minutes). Similarly, the time needed to bring out renal stone was lengthened in the group who had prior history of failed SWL (71.5 vs. 62.3 minutes). In contrast to the observations made by Yuruk E et al.^10^ in another study done by Zhong W et al.^11^ it took significantly longer operative time in SWL failed group (95.8 vs. 80.6 minutes). They noticed inferior clearance of renal stones in group with failed SWL history (83.9% versus 93.4%) which was statistically significant. We had similar success rate among both groups. In present study, the time to create access to pelvicalyceal system was significantly longer in Group-I (previous history of SWL) as compared to Group-II (p value=0.003, Table-II). Transfusion requirements were not dissimilar among their studied groups.^11^ Other complications including fever and peri-renal extravasation were not statistically significant. In present study, the complications were mostly Grade 1 and 2 and were similar across the two groups (Table-III).

Yesil S et al. observed higher frequency of renal vascular injury in subjects who had previous history of open renal stone procedures.^14^ In another study by Türk H et al, prior SWL procedures on the same side kidney did not impact attainment of PCNL success rate, operative time, frequency of adverse surgical outcomes and in hospital stay after surgery.^15^ Although, bleeding was usual in those patients who had previously undergone SWL for renal stones. Operative time and amount of bleeding tend to be higher owing to clinging of calculi fragments in scarred tissues of the renal pelvicalyceal system.^16^-^18^ Reddy SV et al.^19^ mentioned that PCNL can be undertaken with safety in those who had prior surgical history including open stone surgery or PCNL. Avoidance of bumpy handling of nephroscope has a vital role in reducing the frequency of gross complications in patients who had previous open renal surgery.^18^-^22^ Retroperitoneal scarred tissue results in diminished kidney mobility, and reckless handling of nephroscope build a forceful torque that may result in renal lacerations and resultant bleed; at times even major vascular complications.^18^-^22^

### Limitations

This study has few limitations such as a single center study and retrospective in nature. Number of patients having history of SWL was small. In previous studies stones were not categorized according to Guy’s Stone scoring system, which we did. Guy’s stone scores were similar between the two groups. Prospective multicenter studies have not been done regarding this subject.

## CONCLUSION

Those patients who have history of unsuccessful SWL before undertaking the PCNL procedure have almost similar stone free rates and frequency of complications as those observed in SWL naïve cases of PCNL. Moreover, prior history of unsuccessful SWL has no significant impact on operative time and hospital stay.

### Authors’ Contribution:

**NI, SI:** Conceived, designed and did statistical analysis & editing of manuscript, is responsible for integrity of research.

**NI, SI, NZ, AKA:** Did data collection and manuscript writing.

**NI, NZ, AKA:** Did review and final approval of manuscript.

**NI:** Author responsible for accuracy or integrity of the work.
